# Advances in the synthesis of β-alanine

**DOI:** 10.3389/fbioe.2023.1283129

**Published:** 2023-10-26

**Authors:** Peng Song, Xue Zhang, Shuhua Wang, Wei Xu, Feng Wei

**Affiliations:** ^1^ College of Life Sciences, Liaocheng University, Liaocheng, China; ^2^ Shandong Aobo Biotech Co, Ltd., Liaocheng, China

**Keywords:** β-alanine, chemical synthesis, biological synthesis, enzymatic conversion, whole-cell synthesis

## Abstract

β-Alanine is the only naturally occurring β-type amino acid in nature, and it is also one of the very promising three-carbon platform compounds that can be applied in cosmetics and food additives and as a precursor in the chemical, pharmaceutical and material fields, with very broad market prospects. β-Alanine can be synthesized through chemical and biological methods. The chemical synthesis method is relatively well developed, but the reaction conditions are extreme, requiring high temperature and pressure and strongly acidic and alkaline conditions; moreover, there are many byproducts that require high energy consumption. Biological methods have the advantages of product specificity, mild conditions, and simple processes, making them more promising production methods for β-alanine. This paper provides a systematic review of the chemical and biological synthesis pathways, synthesis mechanisms, key synthetic enzymes and factors influencing β-alanine, with a view to providing a reference for the development of a highly efficient and green production process for β-alanine and its industrialization, as well as providing a basis for further innovations in the synthesis of β-alanine.

## 1 Introduction

β-Alanine is the only β-amino acid found in nature and is widely used in pharmaceutical, food, chemical and environmental applications. Many industrially important compounds, such as 3-hydroxypropionic acid (Borodina et al., 2015), poly 3-hydroxypropionate (Lacmata et al., 2017), pantothenic acid (Tadi et al., 2022), carnosine (Kim et al., 2021), pamidronate and balasalazide (Chen et al., 2018), are synthesized using β-alanine as an important precursor or intermediate. In the food industry, β-alanine can be used as a food additive to improve flavor and as a nutritional supplement to improve health (Hooshmand et al., 2019); for athletes, it can significantly increase the carnosine content in the body and thus enhance athletic ability (Matthews et al., 2019). In the feed industry, the addition of β-alanine to feed can regulate the muscle growth of livestock and poultry, improve the antioxidant capacity of muscle, and improve the quality of meat products (Qi et al., 2018). In addition, it can also be directly used in the production of poly-β-alanine, which can be applied in cosmetics, water purification and construction (Steunenberg et al., 2013).

Currently, chemical and biological methods are used in industry to produce β-alanine. The reaction conditions of the chemical method are extreme, including high temperatures and pressures, strongly acidic and strong alkaline conditions, etc., producing more byproducts and requiring high energy consumption (Fujii et al., 1997; Luo et al., 2005). Biological methods (divided into enzymatic conversion and whole-cell synthesis), with their low production cost, mild reaction conditions, high safety and green nature, have become the main research direction. Enzymatic production of β-alanine has strong substrate specificity, is a simple process, and is environmentally friendly, and the product is easy to separate and purify (Chen et al., 2018). However, this method has problems, such as low enzyme activity, susceptibility to organic inactivation, and substrate inhibition. To address these problems, researchers have mainly adopted codon optimization and heterologous expression to improve the expression of related enzymes and site-directed mutagenesis to improve the catalytic stability and efficiency of the enzymes. At the same time, enzyme cascade reactions and other methods have been used to improve the yield of β-alanine by utilizing low-cost raw materials and increasing the β-alanine production rate (Wang et al., 2020; Yuan et al., 2022; Zhou et al., 2022). Whole-cell synthesis of β-alanine, although associated with a high abundance of substrate sources and easy to perform, involves complex microbial metabolic pathways and many byproducts, and the product is difficult to separate and purify. To address the above issues, researchers have mainly focused on systematic modification of the β-alanine synthesis pathway, forcing the host cell metabolism to flow efficiently to the synthesis of β-alanine and thus facilitating whole-cell synthesis by reducing byproduct formation and improving the conversion efficiency (Ghiffary et al., 2022; Yang et al., 2023).

In recent years, the market demand for β-alanine increased annually. β-Alanine is one of the 12 most promising three-carbon platform compounds worldwide (Steunenberg et al., 2013). According to the latest Global Info Research study, the global β-alanine market size was valued at USD 20 million in 2022 and is forecast to reach a readjusted size of USD 31 million by 2029, with a CAGR of 7.0% during the review period (Global Info Research, 2023). In this paper, we provide a comprehensive review on progress in research on the synthesis methods and processes, metabolic engineering, regulatory mechanisms and key enzymes of β-alanine conducted in recent years and focus on the current strategies used by researchers to improve the fermentation pathway, enzymes for catalytic use, and conversion conditions in the biosynthesis pathway to provide a basis for further improvement of the synthetic yield of β-alanine.

## 2 Synthesis of β-alanine

### 2.1 Chemical synthesis

For chemical synthesis of β-alanine, acrylic acid, acrylonitrile, β-aminoacrylonitrile or succinimide can be used as starting material. The traditional process from the last century is still predominant, with acrylic acid and acrylonitrile being the most widely used substrates.

#### 2.1.1 Acrylic acid as substrate

**FIGURE 1:**
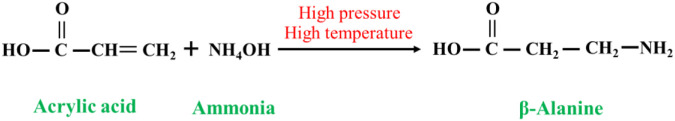
Ammoniation reaction of acrylic acid.

Acrylic acid (or acrylate or acrylate salt) undergoes an ammoniation reaction with ammonia under high-temperature and high-pressure conditions to yield β-alanine, and the ammoniation reaction of acrylic acid proceeds as follows Figure 1. By controlling the reaction conditions or adopting other optimization measures, the final yield of β-alanine in the ammoniation reaction of acrylic acid can be increased up to 90%, with a purity of over 95%. This synthesis method is simple, has a high yield, and is an ideal industrialized production method (Poppelsdorf and Lemon, 1961; Luo et al., 2005).

#### 2.1.2 Acrylonitrile as substrate

**FIGURE 2:**
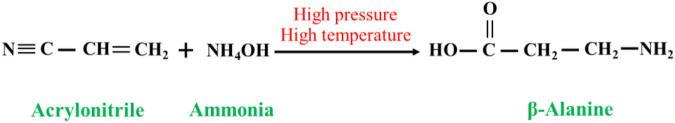
Ammoniation reaction of acrylonitrile.

**FIGURE 3:**
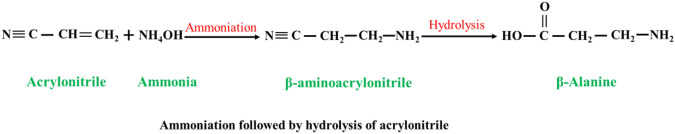
Ammoniation followed by hydrolysis of acrylonitrile.

In this method, acrylonitrile is directly ammoniated or ammoniated and then hydrolyzed to synthesize β-alanine. The direct ammoniation method involves the ammoniation reaction of acrylonitrile and ammonia to produce β-alanine under high-temperature and high-pressure conditions (Figure 2). In the method of ammoniation followed by hydrolysis, acrylonitrile and ammonia react to form the intermediate β-aminoacrylonitrile, which is hydrolyzed to form β-alanine under acidic (or alkaline) conditions (Luo et al., 2005) (Figure 3). The synthesis method is greatly affected by side reactions, the reaction yield is generally not high, and because the hydrolysis process generates many inorganic salts, product purification is more difficult, and the product purity is not high (Ford et al., 1947).

#### 2.1.3 β-aminopropionitrile as substrate

**FIGURE 4:**
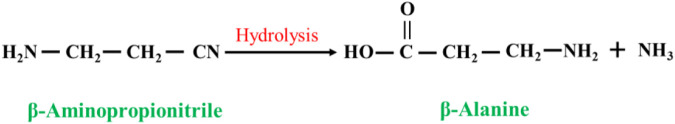
Hydrolysis reaction of β-aminopropionitrile.

In this method, β-aminopropionitrile is used as the substrate, and β-alanine can be synthesized after one-step hydrolysis of the cyano groups (Figure 4). The method can be classified according to the different catalysts used as acid hydrolysis and alkali hydrolysis. Carlson (1943) reported the hydrolysis of β-aminopropionitrile in ammonia at 200 °C for 4 h and a β-alanine yield of approximately 20%. Ford. (1945) studied the hydrolysis of β-aminopropionitrile in barium hydroxide; in 25%–50% barium hydroxide with a reaction temperature of 90–95 °C for 20–30 min, the β-alanine yield was 88%–92%. In practice, liquid alkali (sodium hydroxide or potassium hydroxide) is used to hydrolyze β-aminopropionitrile, and then acid neutralization is used. This method is characterized by a high reaction yield, and the disadvantage is that it produces a large amount of salt.

#### 2.1.4 β-aminopropanol as substrate

**FIGURE 5:**
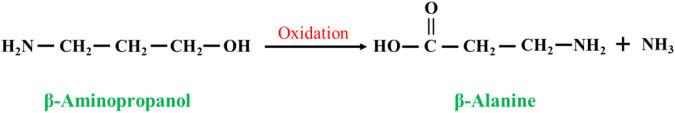
Oxidation reaction of β-aminopropanol.

In the presence of an oxidizing agent, β-aminopropanol is converted to β-alanine by the following reaction (Figure 5). Fujii et al. (1997) oxidized β-aminopropanol to β-alanine at high temperature (100 °C–210 °C) and high pressure (5–50 kg/cm^2^) in alkaline solution using copper-containing catalysts with high yields, few side reactions, and little or no formation of the byproduct methylenedipropionic acid (IDPA), and the substrate conversion was up to 99% with a selectivity of 90%. However, β-aminopropanol is an expensive raw material compared to β-alanine, which is not economically feasible.

#### 2.1.5 Succinimide as substrate

**FIGURE 6:**
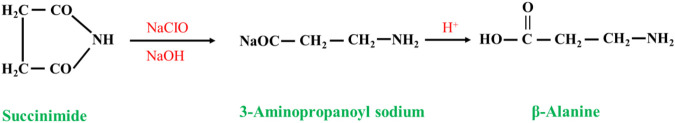
Degradation (Hofmann reaction) of succinimide.

Succinimide undergoes a degradation reaction (Hofmann reaction) in alkaline sodium chlorate solution (a mixture of sodium hypochlorite, sodium hydroxide and sodium carbonate) to form β-alanine via the following reaction (Figure 6). However, this method produces many salts, the product purification is complicated, and the yield is not high (approximately 50%, in terms of succinimide), so it cannot be industrialized (Luo et al., 2005).

Some scholars have also synthesized β-alanine with iminodipropionic acid (Kirk, 1947; Paden, 1947), ethylene cyanhydrin (Kirk and Paden, 1944; Poppelsdorf and Lemon, 1961), or bis(cyanoethyl) amine (Kirk, 1943) as the substrate, but it is difficult to realize industrial production, mainly because of the resource limitations of the substrate itself, the low yield, and the low efficiency of synthesis, among other factors.

### 2.2 Biological synthesis

Biosynthetic methods include enzymatic conversion and whole-cell synthesis. The enzymatic conversion method uses free enzymes in the reaction system to catalyze the direct synthesis of β-alanine. There are two strategies used for whole-cell synthesis: one is to use the microorganisms themselves or engineered bacteria in which β-alanine-related enzymes are heterologously expressed. The bacterium itself is the catalytic medium and is cultured to a certain cell density and then added to the substrate reaction system to catalyze the conversion of the substrate to β-alanine. The other strategy is to utilize the microorganism’s own metabolic system to synthesize β-alanine. The metabolic pathway is modified to balance strain growth and β-alanine production, and β-alanine can be consistently produced as long as nutrients are added and the synthesis conditions are optimized.

#### 2.2.1 Enzymatic conversion

The advantages of the enzymatic synthesis of β-alanine are the mild reaction conditions, high product specificity, low energy consumption, low wear and tear of equipment, high catalytic efficiency of enzymes, simple and easy-to-control process, and green and nonpolluting nature, making it is the most worthwhile method for in-depth development of β-alanine production. Currently, the starting substrates for the enzymatic conversion of β-alanine are mainly β-aminopropionitrile, L-aspartate, fumaric acid, and maleic acid, and the enzymes used in their synthesis and their characteristics are shown in Table 1.

**TABLE 1:** Enzymatic synthesis of β-Alanine.

Substrates	Enzymes	Characteristics
β-Aminopropionitrile	Nitrilases (EC 3.5.5.1)	1) Substrate conversion is high
		2) Reaction steps is less
		3) By-products is less, product is easy to be isolated and purified
L-aspartate	L-aspartate-α-decarboxylase (EC 4.1.1.11, ADC)	4) Substrate is expensive and production costs is high
		5) Enzymes are unstable and prone to be inactivation
Fumaric acid	Aspartate ammonia-lyase (AspAs) (EC 4.3.1.1); L-aspartate-α-decarboxylase (EC 4.1.1.11, ADC)	1) Substrate cost and production cost is low; 2) Intermate L-aspartate accumulates less and inactivation of L-aspartate-α-decarboxylase slows down; 3) The catalytic efficiency of the dual enzyme varies and is not easy to synergize; 4) By-products increase, product is not easy to separated and purified
Maleic acid	Maleic acid isomerase; Aspartate ammonia-lyase (AspAs) (EC 4.3.1.1, AspAs); L-aspartate-α-decarboxylase (EC 4.1.1.11, ADC)	

##### 2.2.1.1 β-Aminopropionitrile as substrate

**FIGURE 7:**
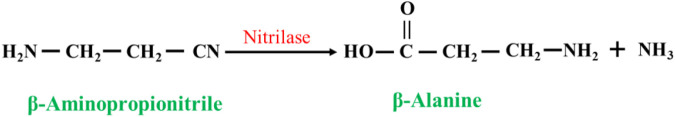
Synthesis of β-alanine from β -aminopropionitrile by nitrilases.

Nitrilases (EC 3.5.5.1) can be used to catalyze the synthesis of β-alanine from β-aminopropionitrile: these enzymes convert the nitrile group in nitrile compounds directly to a carboxyl group to generate carboxylic acids (Liang et al., 2008; Han et al., 2015) (Figure 7). However, when the concentration of the substrate is too high, the generation of the byproduct 3-aminoamide in the reaction system gradually increases, which is difficult to separate from β-alanine and puts great pressure on the subsequent separation process.

##### 2.2.1.2 L-aspartate as substrate

**FIGURE 8:**
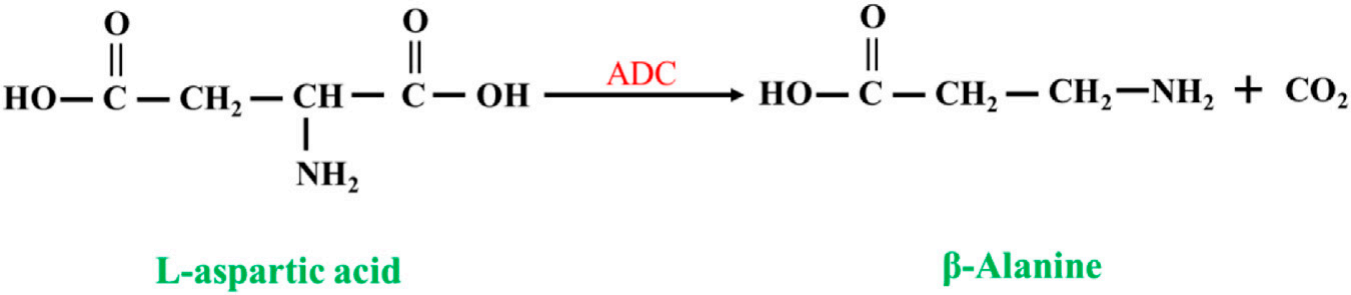
Synthesis of β-alanine from L-aspartate by L-aspartate-α-decarboxylase (ADC).

L-aspartate-α-decarboxylase (EC 4.1.1.11, ADC) catalyzes the synthesis of β-alanine from L-aspartate (Piao et al., 2019; Wang et al., 2022; Hu et al., 2023) (Figure 8). Compared to nitrilases, ADCs have a high substrate concentration and a high conversion rate, so the synthesis of β-alanine is more productive, and at the end of the reaction, the reaction system has a low concentration of byproducts and substrate, which greatly reduces the cost of purification. The disadvantage is that the price of L-aspartate is much higher than that of β-aminopropionitrile.

The ADCs now in use are mainly derived from prokaryotic microorganisms and are mostly heterologously expressed in *Escherichia coli* (*Escherichia coli*). ADCs are mainly derived from *Corynebacterium glutamicum* (*Corynebacterium glutamicum*), *Bacillus subtilis* (*Bacillus subtilis*), *Corynebacterium jeikeium* (*Corynebacterium jeikeium*), *Bacillus tequilensis* (*Bacillus tequilensis*), *and Tribolium castaneum* (*Tribolium castaneum*) (Table 2). To further enhance the catalytic performance of ADCs, the wild-type enzyme is generally modified by means of directed evolution (Pei et al., 2017) and site-directed mutagenesis (Ding and Duan, 2023).

**TABLE 2:** L-aspartate-α-decarboxylase from different microbial sources and their application in β-alanine synthesis.

Microbial sources	Specific activity/(U/mg)	*β*-alanine production (g/L)	References
*E. coli*	∼1.0	—	Pei et al. (2017)
*C. glutamicum*	∼7.8	—	Pei et al. (2017)
*C. glutamicum*	—	26	Wang et al. (2022)
*C. glutamicum*	103.00	12.85	Shen et al. (2014)
*B. subtilis*	—	85.18	Li et al. (2022)
*B. subtilis*	—	166.6	Ghiffary et al. (2022)
*B. subtilis*	∼8.3	∼2.5	Pei et al. (2017)
*B*. *aryabhattai*	33.9	128.67	Ding and Duan (2023)
*T. castaneum*	4.83	143.00	Liu et al. (2019)
*C*. *jeikeium*	0.65	—	Mo et al. (2019)
*B. tequilensis*	—	123.30	Feng et al. (2019)
*T. castaneum*	0.27	—	Ye et al. (2019)
*T. castaneum*	7.046	170.5	Yu et al. (2020)

##### 2.2.1.3 Fumaric acid as substrate

**FIGURE 9:**
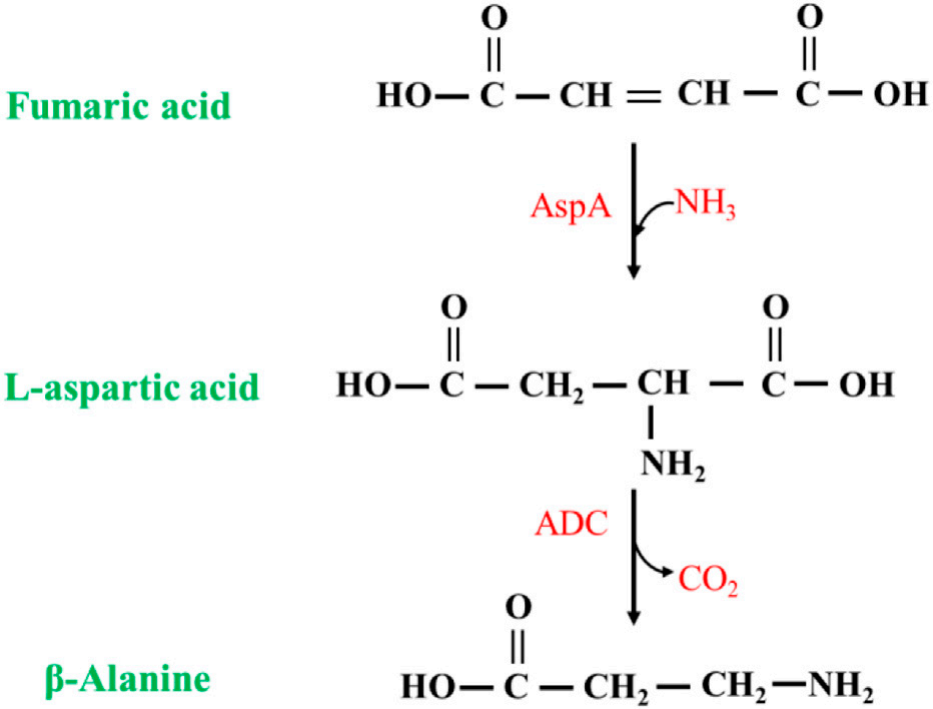
Synthesis of β-alanine from fumaric acid by AspA and ADC cascade reaction.

The synthesis of β-alanine using fumaric acid as a substrate requires two enzymes: aspartate ammonia-lyase (AspA) (EC 4.3.1.1) and an ADC cascade (Figure 9). AspAs catalyzes the generation of L-aspartate from fumaric acid, and ADCs catalyze the conversion of L-aspartate to β-alanine, realizing the synthesis of β-alanine using fumaric acid, which is much less expensive than L-aspartate as a substrate. Moreover, this method has potential for industrial application. Fumaric acid can also be synthesized from maleic acid (via maleic acid isomerase), further expanding the availability of raw materials (Jiao et al., 2017).

The reported sources of AspAs and ADCs in dual-enzyme synthesis are shown in Table 3: the most commonly used AspA is from *E. coli* due to its high catalytic activity and tolerance to high concentrations of substrate. The exploration of the dual enzyme cascade method for the preparation of β-alanine mainly focuses on the co-catalysis of AspA and ADC. The two enzymes have different enzyme activities and catalytic rates, and the dosage of the two enzymes needs to be modulated to obtain the optimal spatiotemporal conversion rate. Another strategy to further simplify the cascade reaction is to co-express the two enzymes in *E. coli* (Qian et al., 2018).

**TABLE 3:** Microbial sources of AspAs and ADCs in dual-enzyme synthesis of β-alanine.

Enzymes	Microbial sources	*β*-alanine production (g/L)	References
AspA	*E. coli*	80.4	Qian et al. (2018)
ADC	*C*. *glutamicum*		
AspA	*E. coli*	118.6	Qian et al. (2020)
ADC	*B*. *subtilis*		
AspA	*B*. *megaterium*	11.68	Tadi et al. (2022)
ADC	*B*. *subtilis*		
AspA	*E. coli*	8.02	Gao et al. (2017)
ADC	*C*. *glutamicum*		

ADC is a key enzyme in the enzymatic synthesis of β-alanine and is required when L-aspartate and fumaric acid are used as substrates. ADCs are classified into two types: one is widely found in prokaryotes and belongs to the pyruvoyl group-dependent enzyme type, with the pyruvoyl group as the active center, and these enzymes are mechanistically inactivated; the other is mostly found in eukaryotes and belongs to the pyridoxal phosphate (PLP)-dependent enzyme type, requiring the addition of PLP as a cofactor in the actual catalytic process (Hu et al., 2023). PLP is expensive, and its use should be avoided as much as possible in industrial production. So, for β-alanine synthesis, a pyruvoyl group-dependent ADC is generally used, which may lead to proton misincorporation during the acceptance of a proton and reformation of the active center of the pyruvoyl group during the reaction, thus changing the pyruvoyl group to an inactive alanyl group, leading to irreversible inactivation of the ADC; this inactivation appears to be inevitable. However, this enzyme inactivation can be weakened by site-directed mutagenesis of the enzyme to increase the correct binding of protons to the enzyme’s active center and to increase the stability of the enzyme’s protein structure. Successful modification of this enzyme has been reported, for example, BsADC of *B. subtilis*, with the E56S mutation (Zhang et al., 2018); an ADC originating from P. dacunhae, with the E88R mutation (Hao et al., 2022); and BsPanD from *B. subtilis*, with the I46V, I88M, K104S, and I126* mutations (Qian et al., 2020), were all successfully improved by the mutations.

In addition to the modification of the catalytic stability of ADC, modification of the self-cleavage efficiency of the enzyme is of high priority. ADC is initially translationally synthesized as an inactive protein (π-protein). Subsequent self-cleavage of the π-protein removes the propeptide, resulting in an active protein form with a β-protein structural domain at the C-terminus and an α-protein structural domain containing an active pyruvoyl group at the N-terminus (Zhao et al., 2022). It was found that the conserved amino acid sequence of low-efficiency self-cleavage ADCs is EGSCA, the conserved amino acid sequence of high-efficiency self-cleavage ADCs is VGSIT, and the conserved amino acid sites near the self-cleavage site, V23, I26, T27, and E56, are important for the efficiency of self-cleavage (Mo, 2019). It is possible to increase the self-cleavage efficiency of ADCs by mutation of these sites, thereby increasing the active protein content and enhancing enzyme activity. Increasing the tolerated substrate concentration, which can increase the yield and reduce wastewater discharge, is an important factor for industrial applications, and the tolerated substrate concentration was dramatically increased by the *C. glutamicum* ADC R12V and Q17A mutations (Wang et al., 2022).

#### 2.2.2 Whole-cell synthesis

As mentioned above, whole-cell synthesis is subdivided into two forms: in one, the microorganism itself, or an engineered bacterium, heterologously expresses enzymes that synthesize β-alanine. The other involves continuous synthesis of β-alanine during the growth and reproduction of the bacterium, which uses its own metabolic pathway to continue synthesizing β-alanine.

##### 2.2.2.1 Whole-cell synthesis of β-alanine by wild-type microorganisms or engineered bacteria which produce relevant enzymes

Wild-type microorganisms produce relevant enzymes that can synthesize β-alanine, or engineered bacteria catalyze the synthesis of β-alanine from relevant substrates after heterologous expression of nitrilases, ADCs, or AspAs. The amount of β-alanine naturally synthesized by wild-type microorganisms is very limited, and engineered bacteria are usually constructed to enhance β-alanine biosynthesis. Currently, *E. coli* and *B. subtilis* are commonly used to heterologously express related enzymes due to their fast growth, high expression efficiency and ease of operation (Table 4). The advantage of this whole-cell catalytic method is that there is no need to crush the cells to obtain the free enzyme, which reduces the handling involved in the process. In this reaction system, the bacterium is added directly, and the reaction is initiated by the addition of substrates such as β-aminopropionitrile, L-aspartate, fumaric acid, or acrylic acid (Table 4). Moreover, the bacterial cells can be recycled (which is convenient for continuous-flow operation), the substrate concentration needed this method is high, and the production efficiency is high.

**TABLE 4:** Whole-cell synthesis of β-alanine from different substrates by engineered bacteria.

Whole cells (engineered bacteria)	Substrates	β-Alanine production (g/L)	References
*E. coli*	L-aspartate	132.0	Feng et al. (2019)
*E. coli*	L-aspartate	24.8	Li et al. (2018)
*E. coli*	L-aspartate	66.4	Fan et al. (2018)
*E. coli*	L-aspartate	12.55	Li et al. (2019)
*E. coli*	L-aspartate	162.0	Piao et al. (2019)
*E. coli*	L-aspartate	215.3	Zhang et al. (2018)
*E. coli*	L-aspartic acid fumaric acid	271.5 200.3	Wang et al. (2020)
*E. coli*	L-aspartic acid	13.2	Li et al. (2019)
*E. coli*	L-aspartic acid	66.4	Fan et al. (2018)
*E. coli*	L-aspartic acid	134.72	Wang et al. (2019)
*E. coli*	L-aspartic acid	71.27	Ye et al. (2019)
*E. coli*	Fumaric acid	300	Chen et al. (2018)
*E. coli*	Fumaric acid	80.4	Qian et al. (2018)
*Rhodococcus* sp. G20	β-aminopropionitrile	—	Liang et al. (2008)

###### 2.2.2.1.1 Whole-cell synthesis of β-alanine from β-aminopropionitrile

Using β-aminopropionitrile as the substrate, β-alanine can be synthesized by one-step hydrolysis of the cyano group. The hydrolysis of 1.0% β-aminopropionitrile was catalyzed for 1 h at 30 °C with a product concentration up to 3.6 g/L using an *Alcaligenes* sp OMT-MY14(Luo et al., 2005). β-aminopropionitrile was also converted to β-alanine by *R. erythropolis* G20 (Liang et al., 2008). Han et al. (2015) developed a tandem reaction strategy that effectively prevented the formation of byproducts as the β-aminopropionitrile concentration increased from 0.6 M to 3.0 M, increasing the possibility of industrial application. A nitrilase from B. japonicum catalyzes the hydrolysis of β-aminopropionitrile to β-alanine at concentrations up to 3.0 mol/L with the formation of 23% 3-aminopropanamide to eliminate the byproduct 3-aminopropanamide, a new nitrilase from P. nitroreducens was cloned and characterized through gene mining. Under the optimal conditions, 3-aminopropanamide was completely hydrolyzed within 12 h. A tandem reaction system was then established to eliminate the byproduct 3-aminopropanamide and increase the production of β-alanine to 90% (isolated yield), with a spatiotemporal yield of 15.02 g/(L.h) (Tao et al., 2016). These results demonstrated that the tandem reaction strategy was an effective method of eliminating the amide byproducts in nitrilase-catalyzed hydrolysis at high substrate concentrations.

The method for the synthesis of β-alanine from organonitrile is milder than the chemical method, but the enzyme is inhibited by the high concentration of substrate, which leads to the inability to synthesize β-alanine with high efficiency. In addition, whole-cell catalysis introduces byproducts, which increases the cost of isolation and purification at a later stage. Therefore, the current level of microbial conversion of β-aminopropionitrile to produce β-alanine is still far from the requirement for industrial production (Gao et al., 2017).

###### 2.2.2.1.2 Whole-cell synthesis of β-alanine from L-aspartate

One-step decarboxylation of L-aspartate catalyzed by ADC to generate β-alanine is one of the most widely studied methods. Screening and mutation of ADCs are of great importance for refining this pathway. Li et al. (2019) heterologously expressed the *B. subtilis panD* gene (encoding ADC) in *E. coli*, and through strategies such as optimization of expression vectors and gene codons, ADC protein production was improved, resulting in a titer of 13.2 g/L β-alanine. Similarly, *E. coli* was used to express the *panD* gene from *Bacillus agave* PanD37. By optimizing the reaction conditions, 100 g/L L-aspartate could be converted to 66.4 g/L β-alanine per hour, and the final substrate conversion rate reached 99.2% (Fan et al., 2018). Zhang et al. (2018) heterologously expressed a panD gene derived from *B. subtilis* in *E. coli* and obtained a E56S mutant with improved enzyme activity. The final *E. coli*/pET28a-E56S strain showed better catalytic stability, reaching a β-alanine concentration of 215.3 g/L within 9 h. Notably, the authors also heterologously expressed mutant panD genes in *B. subtilis* and B. glutamicus as hosts with β-alanine concentrations of 146.7 and 50.4 g/L, respectively. Although this yield was not superior to that of *E. coli*, *B. subtilis* and B. glutamicus have better industrial potential as food-safe strains and deserve further investigation.

Eukaryotic ADCs have received attention due to the general lack of activity of prokaryotic ADCs. Enzymatic studies have shown that they have twice the specific activity of *C. glutamicum*-derived ADCs but suffer from thermal instability (Gao et al., 2017). Multiple mutants of an ADC from the eukaryote *T. castaneum* were heterologously expressed in *E. coli*. Then, a K221R mutant was obtained with improved enzyme activity and stability and was used in whole-cell synthesis. The yield of β-alanine was 134.72 g/L after 23 h of conversion, the spatiotemporal conversion rate was 5.86 g/L/h, and the molar conversion rate was 94.52% (Wang et al., 2019). The research team further modified the *T. castaneum*-derived ADC and screened the K49R mutant with an optimal temperature of 42 °C, which showed significantly higher substrate affinity, 1.7-fold higher thermal stability and 1.8-fold higher catalytic efficiency compared with the wild type. Under the optimized conditions, the concentration of β-alanine reached 71.27 g/L after 11 h, indicating that the insect-derived ADC variant also has great potential for industrial production (Ye et al., 2019).

###### 2.2.2.1.3 Whole-cell synthesis of β-alanine from fumaric acid

Whole-cell catalyzed synthesis of β-alanine using fumaric acid as a substrate involved simultaneous overexpression of aspA and panD in engineered strains. Because of the different catalytic efficiencies of the two enzymes, the key to realizing such a cascade reaction is to balance the expression of the two enzymes in pursuit of a more efficient transformation. Qian et al. (2018) co-expressed the aspA gene from *E. coli* and the panD gene from C. glutamicum in *E. coli*. The production of ADC was enhanced by increasing the gene copy number. The final whole-cell catalyst provided 80.4 ± 1.6 g/L β-alanine in a 5-L reactor with a conversion rate of 95.3% ± 1.6%. In the same year, Chen et al. (2018) synthesized β-alanine using the inexpensive fumaric acid and ammonia as substrates by co-expressing aspA and panD in the same vector with immobilized enzymes. Under optimal conditions, fumaric acid, at a high substrate concentration of 300 g/L, was completely converted to β-alanine. There was no accumulation of the intermediate product L-aspartate in the whole reaction system, which not only simplified the purification but also reduced the production cost. This result suggests that dual enzyme catalysis is a feasible option for industrial applications.

###### 2.2.2.1.4 Whole-cell synthesis of β-alanine from acrylic acid

The acrylic acid metabolic pathway did not originally exist in microorganisms themselves, but certain microorganisms can catalyze the conversion of acrylic acid to β-alanine. The reaction kinetics and process of the ammoniation of acrylic acid to β-alanine were investigated. Although ammonification of acrylic acid to β-alanine is reversible, the reaction kinetics analysis and process simulation showed that the method is feasible (Zhang et al., 1996). Since acrylic acid is relatively inexpensive, the production of β-alanine using acrylic acid as a substrate can be accomplished in a short period with little loss of raw materials and products. This method has obvious economic benefits and deserves further in-depth exploration.

##### 2.2.2.2 Whole-cell synthesis of β-alanine by microbial fermentation

In this method, the microorganism’s own metabolic pathway is utilized to produce β-alanine. β-Alanine is synthesized by microbial fermentation using glucose, glycerol, etc., as carbon sources. Compared to enzymatic conversion or whole-cell synthesis by engineered bacteria, microbial fermentation can be used to synthesize β-alanine from sustainable and environmentally friendly biobased feedstocks (glucose, glycerol, oils, etc.) without having to resort to petroleum-based chemical feedstocks, which has been the most popular research direction in recent years (Miao et al., 2021; Tadi et al., 2022; Yuan et al., 2022). Since *E. coli* has a well-defined genetic background and is easy to cultivate, most researchers have used it as a host for the construction of the β-alanine biosynthesis pathway (Table 5).

**TABLE 5:** Whole-cell synthesis of β-alanine by microbial fermentation.

Whole cells (microbial fermentation)	Carbon source	β-Alanine production (g/L)	References
*E. coli*	Glucose	43.12	Zou et al. (2020)
*E. coli*	Glucose	32.3	Song et al. (2015)
*E. coli*	Glucose	4.7	Gao and Qiu (2007)
*E. coli*	Glycerol	5.0	Liang et al. (2017)
*E. coli*	Glucose	37.7	Piao et al. (2019)
*E. coli*	Glucose	—	Liang et al. (2008)
*E. coli*	Glucose	43.94	Wang et al. (2021)
*E. coli*	Fatty acid	72.05	Miao et al. (2022)
*E. coli*	Glucose	85.18	Li et al. (2022)
*E. coli*	Glycerol	37.9	Xu et al. (2021)
*E. coli*	Glucose	34.8	Yuan et al. (2022)
*E. coli*	Glucose	52.61	Zhou et al. (2022)
*E. coli*	Glycerol	6.65	Xu et al. (2021)
*E. coli*	Glucose	11.9	Hu et al. (2023)
*P. pastoris*	Methanol	5.6	Miao et al. (2021)
*B. megaterium*	Glucose	17.60 ± 0.13	Tadi et al. (2022)

**FIGURE 10:**
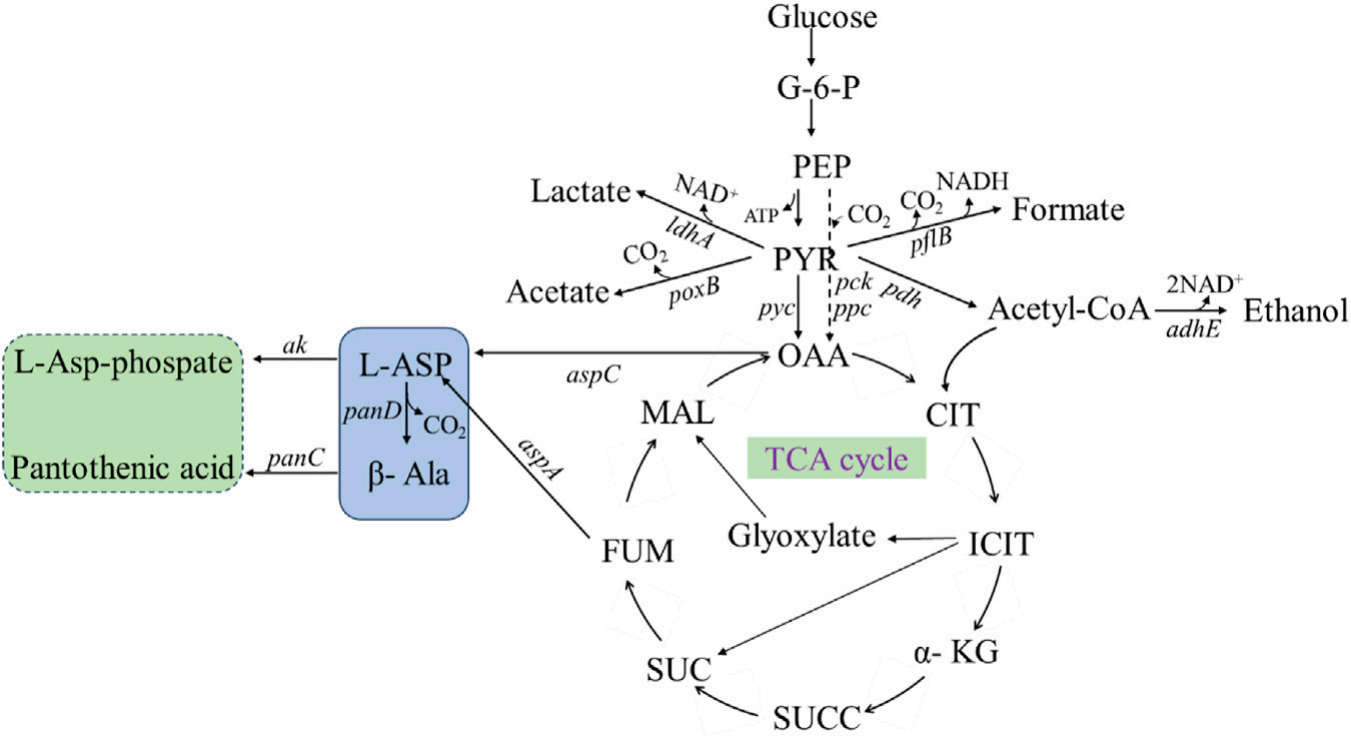
β-Alanine biosynthesis metabolic pathways. PEP: phosphoenolpyruvate; G-6-P: Glucose 6 phosphate; PYR: pyruvate; OAA: oxaloacetate; CIT: citric acid; ICIT: isocitric acid; α-KG: α- Ketoglutaric acid; SUCC: succinyl CoA; SUC: succinic acid; FUM: fumaric acid; MAL: malic acid; L-ASP: L-aspartate; β-Ala: β-alanine; *adhE:* ethanol dehydrogenase; *pflB:* pyruvate formate-lyase; *ldhA:* lactic dehydrogenase; pdh: pyruvate dehydrogenase; *poxB*: pyruvate Oxidase; *pyc*: pyruvate decarboxylase; *pck*: phosphoenolpyruvate carboxykinase; *ppc*: phosphoenolpyruvate carboxylase; *ak*: aspartokinase; *aspC*: aspartate aminotransferase; *aspA*: aspartate ammonia-lyase; *panD*: L-aspartate-α-decarboxylase; *panC*: pantothenate synthetase.

The production of β-alanine by microbial fermentation is mainly dependent on the synthesis pathway of β-alanine in organisms (Figure 10), which is as follows: glucose is transported from the extracellular to intracellular space, catalyzed by glucokinase to produce glucose-6-phosphate (G-6-P) and then phosphoenolpyruvate (PEP). PEP undergoes three reactions for synthesis of β-alanine: 1. PEP is catalyzed by phosphoenolpyruvate carboxykinase and phosphoenolpyruvate carboxylase to oxaloacetate (OAA), which is then catalyzed by aspartate aminotransferase to form L-aspartate (L-Asp), and ADC catalyzes the synthesis of β-alanine from L-aspartate. 2. PEP continues to produce pyruvate (PYR), which in the presence of pyruvate carboxylase generates OAA, which again enters the β-alanine synthesis pathway. 3. PEP generates acetyl coenzyme A (acetyl-CoA) under the action of pyruvate dehydrogenase, which enters the tricarboxylic acid cycle (TCA-cycle), and the intermediate product, fumaric acid, can be catalyzed by AspA to generate L-aspartate. Then, L-aspartate is converted to β-alanine under the action of ADC.

The amount of β-alanine naturally synthesized by wild-type strains is very limited, and engineered strains are usually constructed to enhance β-alanine biosynthesis using metabolic regulation. The commonly used strategies include increasing precursor synthesis, decreasing bypass metabolism, reducing product catabolism, and balancing NADH/NAD^+^.

In the fermentation and transformation process of engineered strains, intermediate metabolites inevitably flow to some irrelevant metabolic pathways, producing unnecessary carbon losses. At this time, it is necessary to weaken the byproduct metabolic pathway while not affecting the normal growth and metabolism of the cells and strengthen the metabolic pathway of β-alanine production to maximize the metabolic flow toward β-alanine production. After the substrate is ingested by the engineered bacteria, it undergoes a series of metabolic processes and eventually enters the TCA cycle in the form of PEP or acetyl-CoA. Currently, it is common to overexpress or (and) optimize the expression of the *ppc* gene using a synthetic promoter and RBS sequences to efficiently convert PEP to oxaloacetate, which significantly increases β-alanine production (Zou et al., 2020; Tunckanat et al., 2022; Dobrowolski et al., 2020; Parthasarathy et al., 2019; Piao et al., 2019; Xu et al., 2021; Song et al., 2015).

In addition to enhancing the metabolic conversion of phosphoenolpyruvate to oxaloacetate, deleting or increasing related metabolic pathways is also a commonly used method to increase oxaloacetate content. In the fermentation and transformation processes of engineered bacteria, lactic dehydrogenase can catalyze the production of lactic acid from pyruvate, pyruvate formate-lyase causes carbon loss, and ethanol dehydrogenase promotes the production of ethanol. Removing *ldhA*, *pflB*, and *adhE* from *E. coli* can further increase the biosynthesis of oxaloacetate (Zou et al., 2020).

The precursor of β-alanine, L-aspartate, is mainly derived from oxaloacetate and fumarate in the TCA cycle, so the efficient synthesis of these two precursors is particularly important in the preparation of β-alanine in high yield. The use of a strong promoter for overexpression of *aspA* allows efficient conversion of fumaric acid to L-aspartate (Song et al., 2015; Zou et al., 2020). L-aspartate is the precursor of many metabolites. In addition to enhancing the main metabolic synthesis of L-aspartate, some of its byproduct pathways can also be appropriately downregulated. Downregulation of the expression of the *akⅠ* and *akⅢ* genes can reduce the unnecessary consumption of L-aspartate without inhibiting the growth of the engineered bacteria (Wang et al., 2021). The introduction of *panD* genes from different sources to catalyze the decarboxylation of L-aspartate to generate β-alanine is the final core step in the metabolic pathways of engineered bacteria.

Currently, the common sources of *panD* are *C. glutamicum* (Song et al., 2015), *B. subtilis* (Piao et al., 2019), *T. castaneum* (Yu et al., 2020), and *B. tequilensis* (Feng et al., 2019). During heterologous expression of *panD* genes from different sources, the promoter of *panD* can be optimized to further increase the yield of β-alanine (Zou et al., 2020). The β-alanine yield can also be further improved by increasing the copy number of *panD*, but the overexpression of *panD* may impose a considerable metabolic burden on the bacterium, which would reduce the β-alanine yield (Miao et al., 2021). β-Alanine metabolism would be accompanied by product accumulation, which may inhibit the growth of the engineered bacteria, and the study proved that when the β-alanine yield reaches 80 g/L, the growth of the strain is extremely adversely affected. The β-alanine transporter (encoded by the *cycA* gene) belongs to the amino acid transporter family, and the growth inhibition of engineered bacteria by β-alanine can be eliminated by deleting *cycA* from the host by using the CRISPR/Cas9 system to avoid β-alanine uptake by the engineered bacteria (Miao et al., 2021; Wang et al., 2021). Of course, knockdown of the *panC* gene and appropriate addition of pantothenic acid to reduce the conversion of β-alanine is also a strategy to increase β-alanine concentration.

NAD(P)H is one of the key cofactors of the metabolic network and plays an important role in the biochemical reactions and physiological functions of amino acid-producing strains. Manipulation of the availability and form of NAD(P)H is an efficient and simple way to shift carbon flux toward amino acid biosynthesis in industrial strains (Xu et al., 2018). The metabolic process of β-alanine synthesis from glucose is accompanied by NAD^+^/NADH interconversion. Glucose enters the body and participates in the glycolytic pathway, in which glyceraldehyde 3-phosphate is produced to generate NADH. NADH is also released during the subsequent synthesis of acetyl-CoA from pyruvate, and NADH is consumed during the subsequent synthesis of L-aspartate from oxaloacetate. Overexpression of bicarbonate transporter and carbonic anhydrase genes enhances CO_2_/bicarbonate assimilation and introduces a cofactor self-contained system, aspartate dehydrogenase, which regenerates NAD^+^ and the NAD^+^ regenerated by this system can be utilized by aspartate aminotransferase to drive the L-aspartate generation reaction more efficiently and increase β-alanine production (Liang, 2017). Knockdown of the *ldhA* gene to balance NADH coenzyme metabolism can effectively increase β-alanine production (Zhou et al., 2016).

Glucose is the most common substrate for the preparation of β-alanine by fermentation, but the substrate suffers from carbon loss and low conversion efficiency. In recent years, Li et al. (2022) introduced an ADC from *B. subtilis* into *E. coli* to construct an initial β-alanine-producing strain. The production of β-alanine was obviously increased by improving the metabolic flux and reducing carbon loss by rerouting fluxes of the central carbon metabolism. Finally, fed-batch bioprocess optimization strategies were used to improve β-alanine production to 85.18 g/L with 0.24 g/g glucose yield and 1.05 g/L/h productivity in fed-batch fermentation.

Miao et al. (2021) utilized *Pichia pastoris* for β-alanine production using methanol as a carbon source and obtained 5.60 g/L β-alanine. Miao et al. (2022) synthesized β-alanine by modulating the glyoxylate-TCA cycle pathway using fatty acids as raw materials and finally obtained 72.05 g/L β-alanine with a conversion rate as high as 1.24 g/g. This method solved the problems of carbon loss and inhibition of cell growth by using fatty acids as raw materials.

The fermentation method relies on the growth and reproduction of microorganisms, which requires a certain duration, so the production efficiency is inevitably low. Among the metabolic pathways of microorganisms, L-aspartate is the precursor of more than 10 metabolites, which involves protein synthesis and the production of other L-aspartate family amino acids (Li et al., 2017), as well as the reaction of other intermediate metabolites in the cell, so unnecessary consumption often occurs in the production of β-alanine by aerobic fermentation. At the same time, due to the extremely large number of intermediates in the microbial metabolic stream, it is difficult to determine what the bottleneck is for the efficient production of β-alanine, making the application of the fermentation method to the industrialized production of β-alanine more difficult.

## 3 Prospects and outlook

β-Alanine is widely used in several fields. The production of β-alanine by chemical methods is characterized by harsh reaction conditions, the formation of many byproducts and high energy consumption. The development of green and environmentally friendly biological methods for the preparation of β-alanine is steadily progressing, and this method has gradually become the mainstream production method for β-alanine. Currently, the preparation of β-alanine by biological methods mainly includes whole-cell synthesis (including fermentation synthesis) and enzymatic synthesis. To date, a β-alanine production level of 300 g/L has been achieved using the whole-cell catalytic method (Chen et al., 2018). Further screening of higher activity L-aspartate-α-decarboxylase, optimization of bioconversion conditions, and recycling of whole cells using cell immobilization technology will further enhance the efficiency and industrialization of whole-cell catalysis.

For fermentation production of β-alanine, an inexpensive substrate such as glucose, glycerol, fatty acids, xylose, or lignocellulosic hydrolysate can be used as a carbon source, which is both cost effective and environmentally friendly and is in line with the trend of green chemistry and next-generation industrial production. Fermentation methods with yields exceeding 100 g/L β-alanine are considered competitive production methods. At present, the highest yield of β-alanine obtained by the fermentation method is 166.6 g/L (Ghiffary et al., 2022), which is achieved the industrial production level and warrants exploitation. Currently, β-alanine fermentation production strains are mainly dominated by *E. coli*, while the selection of GRAS strains such as *C. glutamicum* and *B. subtilis* is more favorable for the production of food-grade and feed-grade β-alanine. To obtain high-yield β-alanine-producing strains, in the future, it will be necessary to generate β-alanine-tolerant strains by means of genetic engineering to balance the relationship between strain growth and β-alanine synthesis via metabolic pathway modification and to realize low-cost and high-efficiency extraction of the product.

Regarding enzymatic conversion, currently, the main substrates are β-aminoalanonitrile, L-aspartate, fumaric acid and maleic acid. ADC is the key enzyme for most substrates. Considering that this enzyme is always obtained by heterologous expression, site-directed mutagenesis and codon optimization are mostly used to overcome its natural defects. With the development of artificial intelligence, machine learning and deep learning, artificial design of enzyme proteins is gradually becoming a reality. The application of the above new technologies provides new technical means for analysis of the catalytic mechanisms of enzymes and for artificial design. Computer-aided design will efficiently solve the current problems of low catalytic efficiency and easy inactivation of related enzymes (Mazurenko et al., 2020; Wittmann et al., 2021; Derat and Kamerlin, 2022; Quan et al., 2023).

Finally, certain microorganisms can catalyze the conversion of acrylic acid to β-alanine. It will be important to screen and identify the catalytic enzymes in these organisms as soon as possible. From an economic perspective, the synthesis of β-alanine using acrylic acid as a substrate would be a very suitable route. Of course, screening for strains and enzymes that are tolerant to high concentrations of substrates is a constant concern in the development of all the above technologies.

## References

[B1] Borodina I. Kildegaard K. R. Jensen N. B. Blicher T. H. Maury J. Sherstyk S. (2015). Establishing a synthetic pathway for high-level production of 3-hydroxypropionic acid in saccharomyces cerevisiae via β-alanine. Metab. Eng. 27, 57–64. 10.1016/j.ymben.2014.10.003 25447643

[B2] Carlson G. (1943). Preparation of beta-alanine. U.S. Patent No 44849242A. Washington, DC: U.S. Patent and Trademark Office.

[B3] Chen M. Qi Y. Xiao Y. Hua C. Li J. Liang A. (2018). Biocatalytic synthesis of β-alanine from fumaric acid by a two-enzyme system. Bull. Ferment. 47, 231–235.

[B4] Derat E. Kamerlin S. C. L. (2022). Computational advances in protein engineering and enzyme design. J. Phys. Chem. C. B 126, 2449–2451. 10.1021/acs.jpcb.2c01198 35387452

[B5] Ding Q. Duan X. (2023). A high-specific-activity L-aspartate-α-decarboxylase from Bacillus aryabhattai gel-09 and site-directed mutation to improve its substrate tolerance. Appl. Biochem. Biotech. 195, 5802–5822. 10.1007/s12010-023-04360-w 36708489

[B6] Dobrowolski S. F. Alodaib A. Karunanidhi A. Basu S. Holecko M. Lichter-Konecki U. (2020). Clinical, biochemical, mitochondrial, and metabolomic aspects of methylmalonate semialdehyde dehydrogenase deficiency: report of a fifth case. Mol. Genet. Metab. 129, 272–277. 10.1016/j.ymgme.2020.01.005 32151545

[B7] Fan X. Feng Z. Fang M. Zhang J. Chen G. Li L. (2018). Heterologous expression of the Bacillus tequilensis L-aspartate α-decarboxylase in *Escherichia coli* and its high cell density fermentation. Food. Sci. 39, 144–150. 10.7506/spkx1002-6630-201802023

[B8] Feng Z. Zhang J. Chen G. Ge Y. Zhang X. Zhu H. (2019). Extracellular expression of L-aspartate-α-decarboxylase from Bacillus tequilensis and its application in the biosynthesis of β-alanine. Appl. Biochem. Biotech. 189, 273–283. 10.1007/s12010-019-03013-1 30972708

[B9] Ford J. H. (1945). The alkaline hydrolysis of β-aminopropionitrile. J. Am. Chem. Soc. 67, 876–877. 10.1021/ja01221a503

[B10] Ford J. H. Buc S. R. Greiner J. W. (1947). An improved synthesis of β-alanine. III. The addition of ammonia to acrylonitrile at 50-150°^1^ . J. Am. Chem. Soc. 69, 844–846. 10.1021/ja01196a029 20292471

[B11] Fujii T. Takayashiki K. Takasaki S. (1997). Production of beta-alanine salt. Jpn. Pat. No JPH09151168A.

[B12] Gao L. Qiu J. (2007). Research advances in L-aspartate decarboxylase. Ind. Microbiol. 37, 54–59. 10.1016/S1872-2040(07)60059-0

[B13] Gao Y. Liu Z. Liu K. Zhou Z. Cui W. (2017). Biocatalytic access to β-alanine by a two-enzyme cascade synthesis. Chin. J. Biotechnol. 33, 875–879. 10.13345/j.cjb.160416 28876041

[B14] Ghiffary M. R. Prabowo C. P. S. Adidjaja J. J. Lee S. Y. Kim H. U. (2022). Systems metabolic engineering of Corynebacterium glutamicum for the efficient production of β-alanine. Metab. Eng. 74, 121–129. 10.1016/j.ymben.2022.10.009 36341775

[B15] Global Info Research (2023). Global β-Alanine Market 2023 by manufacturers, regions, type and application. Available at: https://www.globalinforesearch.com/reports/993343/alanine .

[B16] Han C. Yao P. Yuan J. Duan Y. Feng J. Wang M. (2015). Nitrilase-catalyzed hydrolysis of 3-aminopropionitrile at high concentration with a tandem reaction strategy for shifting the reaction to β-alanine formation. J. Mol. Catal. B, Enzym. 115, 113–118. 10.1016/j.molcatb.2015.02.007

[B17] Hao M. Cui R. Zhu X. Han L. Zhou Z. Liu Z. (2022). Improving the activity and synergistic catalysis of L-aspartate β-decarboxylase by arginine introduction on the surface. Catal. Sci. Technol. 12, 5281–5289. 10.1039/D2CY00700B

[B18] Hooshmand S. Halabchi F. Hashempour A. Rajabian Tabesh M. Alizadeh Z. (2019). Improving physical activity tolerance in sedentary overweight women under beta-alanine supplementation. Sci. Sport. 34, e217–e223. 10.1016/j.scispo.2018.12.004

[B19] Hu S. Fei M. Fu B. Yu M. Yuan P. Tang B. (2023). Development of probiotic *E. coli* Nissle 1917 for β-alanine production by using protein and metabolic engineering. Appl. Microbiol. Biot. 107, 2277–2288. 10.1007/s00253-023-12477-5 36929190

[B20] Hu Z. Tian Y. Yang J. Zhu Y. Zhou H. Zheng Y. (2023). Research progress of l-aspartate-α-decarboxylase and its isoenzyme in the β-alanine synthesis. World J. Microb. Biot. 39, 42. 10.1007/s11274-022-03483-2 36513951

[B21] Jiao Q. C. Liu J. Z. Wei Y. Zhang W. Wu T. Cao S. (2017). The invention relates to a method for preparing β-alanine by multienzyme coupling with maleic acid as raw material. CN Patent No CN107012180A. Beijing: State Intellectual Property Office of the P.R.C.

[B22] Kim M. Ko Y. J. Jeong D. W. Jeong W. Han S. O. (2021). Ecofriendly synthesis of L-Carnosine in metabolically engineered Corynebacterium glutamicum by reinforcing precursor accumulation. ACS Synth. Biol. 10, 1553–1562. 10.1021/acssynbio.1c00168 34019768

[B23] Kirk P. M. (1943). Preparation of beta-alanine. U.S. Patent No US44848642A. Washington, DC: U.S. Patent and Trademark Office.

[B24] Kirk P. M. (1947). Preparation of beta-alanine. U.S. Patent No US44848942A. Washington, DC: U.S. Patent and Trademark Office.

[B25] Kirk P. M. Paden J. H. (1944). Preparation of beta -alanine. U.S. Patent No US44848742A. Washington, DC: U.S. Patent and Trademark Office.

[B26] Lacmata S. T. Kuiate J. Ding Y. Xian M. Liu H. Boudjeko T. (2017). Enhanced poly(3-hydroxypropionate) production via β-alanine pathway in recombinant *Escherichia coli* . PloS One 12, e0173150. 10.1371/journal.pone.0173150 28253372 PMC5333900

[B27] Li B. Su C. Chao F. Hong H. Zhang C. (2019). Construction and optimization of high-yield β-alanine genetically engineered bacteria. J. Dalian Polytech. Univ. 38, 1–4. 10.19670/j.cnki.dlgydxxb.2019.0101

[B28] Li B. Zhang B. Wang P. Cai X. Chen Y. Yang Y. (2022). Rerouting fluxes of the central carbon metabolism and relieving mechanism-based inactivation of l-Aspartate-α-decarboxylase for fermentative production of β-alanine in *Escherichia coli* . ACS Synth. Biol. 11, 1908–1918. 10.1021/acssynbio.2c00055 35476404

[B29] Li H. Lu X. Chen K. Yang J. Zhang A. Wang X. (2018). β-alanine production using whole-cell biocatalysts in recombinant *Escherichia coli* . Mol. Catal. 449, 93–98. 10.1016/j.mcat.2018.02.008

[B30] Li Y. Wei H. Wang T. Xu Q. Zhang C. Fan X. (2017). Current status on metabolic engineering for the production of L-aspartate family amino acids and derivatives. Bioresour. Technol. 245, 1588–1602. 10.1016/j.biortech.2017.05.145 28579173

[B31] Liang L. Jin S. Xu J. Zheng Y. Shen Y. (2008). Isolation and identification of a bacterial strain G20 capable of β-aminopropionitrile bioconversion to β-alanine. Food Ferment. Ind. 34, 485–488. 10.1210/jcem-66-3-485

[B32] Liang L. Zheng Y. Shen Y. (2008). Optimization of β-alanine production from β-aminopropionitrile by resting cells of Rhodococcus sp. G20 in a bubble column reactor using response surface methodology. Process Biochem. 43, 758–764. 10.1016/j.procbio.2008.03.002

[B33] Liang S. Zhou L. Zhang B. Zhou Z. (2017). Metabolic engineering of *Escherichia coli* for the production of β-alanine. Food Ferment. Ind. 43, 13–18.

[B34] Liang S. S. (2017). Metabolic engineering of *Escherichia coli* for the production of β-alanine. Wuxi: Jiangnan University.

[B35] Liu Z. Zheng W. Ye W. Wang C. Gao Y. Cui W. (2019). Characterization of cysteine sulfinic acid decarboxylase from *Tribolium castaneum* and its application in the production of β-alanine. Appl. Microbiol. Biot. 103, 9443–9453. 10.1007/s00253-019-10139-z 31696283

[B36] Luo J. Xue J. Shen Y. (2005). Synthesis and application of β-alanine. Amino Acids Biot. Res. 27, 52–55.

[B37] Matthews J. J. Artioli G. G. Turner M. D. Sale C. (2019). The physiological roles of carnosine and β-alanine in exercising human skeletal muscle. Med. Sci. Sport Exer. 51, 2098–2108. 10.1249/MSS.0000000000002033 31083045

[B38] Mazurenko S. Prokop Z. Damborsky J. (2020). Machine learning in enzyme engineering. ACS Catal. 10, 1210–1223. 10.1021/acscatal.9b04321

[B39] Miao L. Li Y. Zhu T. (2021). Metabolic engineering of methylotrophic Pichia pastoris for the production of β-alanine. Bioresour. Bioprocess. 8, 89–11. 10.1186/S40643-021-00444-9 PMC10991944 38650288

[B40] Miao Y. Liu J. Wang X. Liu B. Liu W. Tao Y. (2022). Fatty acid feedstocks enable a highly efficient glyoxylate‐TCA cycle for high‐yield production of β‐alanine. Mlife 1, 171–182. 10.1002/mlf2.12006 PMC10989975 38817673

[B41] Mo Q. (2019). Molecular mechanism of the catalytic inactivation of L-aspartate alpha-decarboxylase. Wuxi: Jiangnan University.

[B42] Mo Q. Mao A. Li Y. Shi G. (2019). Substrate inactivation of bacterial l-aspartate α-decarboxylase from Corynebacterium jeikeium K411 and improvement of molecular stability by saturation mutagenesis. World J. Microb. Biot. 35, 62–68. 10.1007/s11274-019-2629-6 30923994

[B43] Paden J. H. (1947). Preparation of beta-alanine. U.S. Patent No US44848842A. Washington, DC: U.S. Patent and Trademark Office.

[B44] Parthasarathy A. Savka M. A. Hudson A. O. (2019). The synthesis and role of β-alanine in plants. Front. Plant Sci. 10, 921. 10.3389/fpls.2019.00921 31379903 PMC6657504

[B45] Pei W. Zhang J. Deng S. Tigu F. Li Y. Li Q. (2017). Molecular engineering of l-aspartate-α-decarboxylase for improved activity and catalytic stability. Appl. Microbiol. Biot. 101, 6015–6021. 10.1007/s00253-017-8337-y 28589224

[B46] Piao X. Wang L. Lin B. Chen H. Liu W. Tao Y. (2019). Metabolic engineering of *Escherichia coli* for production of L-aspartate and its derivative β-alanine with high stoichiometric yield. Metab. Eng. 54, 244–254. 10.1016/j.ymben.2019.04.012 31063790

[B47] Poppelsdorf F. Lemon R. C. (1961). Notes. Improved synthesis of β-alanine. Chem 26, 262–263. 10.1021/jo01060a617

[B48] Qi B. Wang J. Ma Y. Wu S. Qi G. Zhang H. (2018). Effect of dietary β-alanine supplementation on growth performance, meat quality, carnosine content, and gene expression of carnosine-related enzymes in broilers. Poult. Sci. 97, 1220–1228. 10.3382/ps/pex410 29325148

[B49] Qian Y. Liu J. Song W. Chen X. Luo Q. Liu L. (2018). Production of β‐Alanine from fumaric acid using a Dual‐Enzyme cascade. Chemcatchem 10, 4984–4991. 10.1002/cctc.201801050

[B50] Qian Y. Lu C. Liu J. Song W. Chen X. Luo Q. (2020). Engineering protonation conformation of L‐aspartate-α-decarboxylase to relieve mechanism‐based inactivation. Biotechnol. Bioeng. 117, 1607–1614. 10.1002/bit.27316 32096553

[B51] Quan L. Wu T. Lyu Q. (2023). Computational *de novo* protein design: from secondary to primary, then toward tertiary structures. Chem 9, 1625–1627. 10.1016/j.chempr.2023.06.010

[B52] Shen Y. Zhao L. Li Y. Zhang L. Shi G. (2014). Synthesis of β-alanine from l-aspartate using l-aspartate-α-decarboxylase from Corynebacterium glutamicum. Biotechnol. Lett. 36, 1681–1686. 10.1007/s10529-014-1527-0 24737081

[B53] Song C. W. Lee J. Ko Y. S. Lee S. Y. (2015). Metabolic engineering of *Escherichia coli* for the production of 3-aminopropionic acid. Metab. Eng. 30, 121–129. 10.1016/j.ymben.2015.05.005 26057003

[B54] Steunenberg P. Konst P. M. Scott E. L. Franssen M. C. R. Zuilhof H. Sanders J. P. M. (2013). Polymerisation of β-alanine through catalytic ester-amide exchange. Eur. Polym. J. 49, 1773–1781. 10.1016/j.eurpolymj.2013.03.032

[B55] Tadi S. R. R. Nehru G. Limaye A. M. Sivaprakasam S. (2022). High-level expression and optimization of pantoate-β-alanine ligase in Bacillus megaterium for the enhanced biocatalytic production of D-pantothenic acid. J. Food Sci. Tech. 59, 917–926. 10.1007/s13197-021-05093-6 PMC8814086 35153321

[B56] Tadi S. R. R. Nehru G. Sivaprakasam S. (2022). Metabolic engineering of Bacillus megaterium for the production of β-alanine. Biotechnol. Bioproc. E. 27, 909–920. 10.1007/s12257-022-0077-x

[B57] Tadi S. R. R. Nehru G. Sivaprakasam S. (2022). One-pot biosynthesis of 3-aminopropionic acid from fumaric acid using recombinant Bacillus megaterium containing a linear dual-enzyme cascade. Appl. Biochem. Biotechnol. 194, 1740–1754. 10.1007/s12010-021-03783-7 34997447

[B58] Tao Y. Yao P. Yuan J. Han C. Feng J. Wang M. (2016). Efficient biosynthesis of β-alanine with a tandem reaction strategy to eliminate amide by-product in the nitrilase-catalyzed hydrolysis. J. Mol. Catal. B. Enzym. 133, S60–S67. 10.1016/j.molcatb.2016.11.018

[B59] Tunckanat T. Gendron A. Sadler Z. Neitz A. Byquist S. Lie T. J. (2022). Lysine 2,3-aminomutase and a newly discovered glutamate 2,3-aminomutase produce β-amino acids involved in salt tolerance in methanogenic archaea. Biochem. East. 61, 1077–1090. 10.1021/acs.biochem.2c00014 35544775

[B60] Wang C. Ye W. Xue L. Liu Z. Zhou Z. (2019). The impact of analgosedation on mortality and delirium in critically ill patients: a systematic review and meta-analysis. Food. Ferment. Ind. 45, 7–14. 10.1016/j.iccn.2019.06.004 31395447

[B61] Wang J. Ma D. Mai D. Li H. Wang J. Wang X. (2022). β-Alanine production by L-aspartate-α-decarboxylase from Corynebacterium glutamicum and variants with reduced substrate inhibition. Mol. Catal. 522, 112246. 10.1016/j.mcat.2022.112246

[B62] Wang L. Mao Y. Wang Z. Ma H. Chen T. (2021). Advances in biotechnological production of β-alanine. World J. Microb. Biot. 37, 79–11. 10.1007/s11274-021-03042-1 33825146

[B63] Wang L. Piao X. Cui S. Hu M. Tao Y. (2020). Enhanced production of β-alanine through co-expressing two different subtypes of l-aspartate-α-decarboxylase. J. Ind. Microbiol. Biot. 47, 465–474. 10.1007/s10295-020-02285-5 32524454

[B64] Wang P. Zhou H. Y. Li B. Ding W. Q. Liu Z. Q. Zheng Y. G. (2021). Multiplex modification of *Escherichia coli* for enhanced β-alanine biosynthesis through metabolic engineering. Bioresour. Technol. 342, 126050. 10.1016/J.BIORTECH.2021.126050 34597803

[B65] Wittmann B. J. Yue Y. Arnold F. H. (2021). Informed training set design enables efficient machine learning-assisted directed protein evolution. Cell Syst. 12, 1026–1045.e7. 10.1016/j.cels.2021.07.008 34416172

[B66] Xu J. Yang H. Zhang W. (2018). NADPH metabolism: a survey of its theoretical characteristics and manipulation strategies in amino acid biosynthesis. Crit. Rev. Biotechnol. 38, 1061–1076. 10.1080/07388551.2018.1437387 29480038

[B67] Xu J. Zhou L. Yin M. Zhou Z. (2021). Novel mode engineering for β-alanine production in *Escherichia coli* with the guide of adaptive laboratory evolution. Microorg. (Basel) 9, 600. 10.3390/microorganisms9030600 PMC8000549 33803992

[B68] Xu J. Zhou L. Zhou Z. (2021). Enhancement of β-alanine biosynthesis in *Escherichia coli* based on multivariate modular metabolic engineering. Biol. (Basel, Switz. 10, 1017. 10.3390/biology10101017 PMC8533518 34681116

[B69] Xu J. Zhu Y. Zhou Z. (2021). Systematic engineering of the rate-limiting step of β-alanine biosynthesis in *Escherichia coli* . Electron. J. biotechn. 51, 88–94. 10.1016/j.ejbt.2021.03.002

[B70] Yang S. Li J. Meng R. Yu T. Wang Z. Xiong P. (2023). Screening and identification of genes involved in β-alanine biosynthesis in Bacillus subtilis. Arch. Biochem. Biophys. 743, 109664. 10.1016/j.abb.2023.109664 37301357

[B71] Ye W. Q. Xue L. Wang C. Cui W. J. Zhou Z. M. Liu Z. M. (2019). Characterization of enzymatic properties of an insect-derived L-aspartate-α-decarboxylase variant. Food Ferment. Industries 45, 63–67. 10.13995/j.cnki.11-1802/ts.021344

[B72] Yu X. Huang C. Xu X. Chen H. Liang M. Xu Z. (2020). Protein engineering of a pyridoxal-5′-phosphate-dependent l-Aspartate-α-Decarboxylase from *Tribolium castaneum* for β-alanine production. Mol. (Basel, Switz. 25, 1280. 10.3390/molecules25061280 PMC7143960 32178239

[B73] Yuan S. Nair P. H. Borbon D. Coleman S. M. Fan P. Lin W. (2022). Metabolic engineering of *E. coli* for β-alanine production using a multi-biosensor enabled approach. Metab. Eng. 74, 24–35. 10.1016/j.ymben.2022.08.012 36067877

[B74] Zhang K. Lu N. Xu H. (1996). Reaction kinetics and process simulation for producing β-alanine by acrylic acid ammoniation (I): kinetics of the reaction of acrylic acid with aqueous ammonia. Chem. React. Eng. Technol. 12, 160.

[B75] Zhang T. Zhang R. Xu M. Zhang X. Yang T. Liu F. (2018). Glu56Ser mutation improves the enzymatic activity and catalytic stability of Bacillus subtilis l-aspartate α-decarboxylase for an efficient β-alanine production. Process Biochem. 70, 117–123. 10.1016/j.procbio.2018.04.004

[B76] Zhao M. Wang M. Peng L. Liu W. Song X. Liu Z. (2022). Determination of three sites involved in the divergence of L-aspartate-α-decarboxylase self-cleavage in bacteria. Enzyme Microb. Tech. 158, 110048. 10.1016/j.enzmictec.2022.110048 35447535

[B77] Zhou H. Tang Y. Peng J. Wang S. Liu Z. Zheng Y. (2022). Re-designing *Escherichia coli* for high-yield production of β-alanine by metabolic engineering. Biochem. Eng. J. 189, 108714. 10.1016/j.bej.2022.108714

[B78] Zhou L. Deng C. Cui W. Liu Z. Zhou Z. (2016). Efficient L-alanine production by a thermo-regulated switch in *Escherichia coli* . Appl. Biochem. Biotech. 178, 324–337. 10.1007/s12010-015-1874-x 26453031

[B79] Zou X. Guo L. Huang L. Li M. Zhang S. Yang A. (2020). Pathway construction and metabolic engineering for fermentative production of β-alanine in *Escherichia coli* . Appl. Microbiol. Biot. 104, 2545–2559. 10.1007/s00253-020-10359-8 31989219

